# Data of a meta-analysis on pharmacological treatment strategies for lowering prolactin in people with a psychotic disorder and hyperprolactinaemia

**DOI:** 10.1016/j.dib.2020.105904

**Published:** 2020-06-23

**Authors:** Javier Labad, Itziar Montalvo, Alexandre González-Rodríguez, Clemente García-Rizo, Benedicto Crespo-Facorro, José Antonio Monreal, Diego Palao

**Affiliations:** a Department of Mental Health, Hospital Universitari Parc Taulí, I3PT. Sabadell, Barcelona, Spain; b Department of Psychiatry and Legal Medicine, Universitat Autònoma de Barcelona. Cerdanyola, Spain; c Centro de Investigación Biomédica en Red (CIBERSAM), Spain; d Barcelona Clinic Schizophrenia Unit, Hospital Clínic, IDIBAPS. University of Barcelona, Spain; e University Hospital Virgen del Rocío, IBiS, Departament of Psychiatry, University of Sevilla, Sevilla, Spain

**Keywords:** Hyperprolactinaemia, Schizophrenia, Antipsychotics, Aripiprazole, Dopamine agonists, Switching

## Abstract

The data presented in this paper describe supplementary material to the article entitled “Pharmacological treatment strategies for lowering prolactin in people with a psychotic disorder and hyperprolactinaemia: a systematic review and meta-analysis” [1]. Although raw data was published on the original article, additional raw data has been included in the current paper (new tables with socio-demographic and clinical characteristics of the samples of the studies included in the systematic review). Supplementary data also include the PICO scheme of the systematic review, PRISMA checklist, flow diagram, an explanation of the method for obtaining prolactin concentrations from published figures when data was only available in figures, list of the selected studies, risk of bias summary of all five randomized clinical trials evaluating the addition of aripiprazole for lowering prolactin (included in the meta-analysis in the original article). Extra analyses, figures and R code of the meta-analysis have been also included. Meta-analysis of randomized clinical trials (RCTs) considering aripiprazole addition for lowering prolactin in people with a psychotic disorder and hyperprolactinaemia were conducted with two softwares: 1) R and the metaphor package (for the meta-analysis of the primary outcome [prolactin reduction]); 2) MedCalc version 18.11 (for the meta-analysis of the secondary outcome [withdrawal rates]). Data from a sensitivity analysis (repeating the meta-analysis with only placebo-controlled RCTs) has been also included in the current article.

**Specifications Table**

**Table:** 

**Subject**	Psychiatry and Mental Health; Endocrinology, Diabetes and Metabolism
**Specific subject area**	Management of antipsychotic-induced hyperprolactinaemia
**Type of data**	Table Figure Box Code List
**How data were acquired**	Four electronic bibliographic databases were searched: PubMed, Scopus, PsycINFO and ClinicalTrials.gov. The following search strategy was used: prolactin AND (switch* OR aripiprazole OR bromocriptine OR cabergoline OR "dopamine agonist" OR metformin) AND (schizophrenia OR schizoaffective OR psychosis OR psychotic OR bipolar*). Language was restricted to those articles written in English, Spanish, German or French. Studies published between January 1980 and March 2020 were considered for inclusion. The systematic review contained trials including patients with psychotic disorders (schizophrenia, schizoaffective disorder, bipolar disorder, brief psychotic disorder, delusional disorder, or psychotic disorder not otherwise specified) who had hyperprolactinaemia. Clinical trials and observational studies were included if they assessed the efficacy of any of the following four therapeutic options for lowering prolactin (switching antipsychotic treatment, adding aripiprazole, adding other dopamine agonists [e.g., cabergoline, bromocriptine], or adding metformin) and had information on prolactin levels at baseline and after follow-up that would allow the calculation of the effect size for the reduction in prolactin concentrations. Exclusion criteria were case reports or studies of less than 5 cases, and studies assessing prolactin changes in psychotic patients for whom the main reason for the therapeutic strategy was not hyperprolactinaemia (e.g., switching in treatment-resistant patients). Although the systematic review included clinical trials and observational studies, only randomized clinical trials (RCTs) were considered for conducting a meta-analysis. The primary outcome was defined as the reduction in prolactin concentrations. The software R and the package metafor were used for conducting the meta-analysis on the primary outcome (reduction in prolactin levels). Hedges g’ was used as the effect size for prolactin reduction. An additional meta-analysis for withdrawal rates in placebo-controlled RCTs was performed with MedCalc (version 18.11).
**Data format**	Raw data of prolactin concentrations and withdrawal rates are included in the original article Additional raw data on the current article Analyzed Filtered Methodological explanations
**Parameters for data collection**	We aimed to identify clinical trials and observational studies considering four therapeutic options for lowering prolactin in people with a psychotic disorder and hyperprolactinaemia: switching antipsychotic treatment, adding aripiprazole, adding other dopamine agonists (e.g., cabergoline, bromocriptine), or adding metformin.
**Description of data collection**	The PRISMA group guidelines were followed. A standardised, pre-piloted form was used to extract data from the included studies for assessment of the study quality and evidence synthesis. Two review authors extracted data independently, and discrepancies were identified and resolved through discussion (with two additional authors when necessary). Missing data were requested from study authors. In a few cases in which data were only available in figures (either as individual concentrations or aggregated with mean and standard deviation), we extracted this information from figures using the procedure explained in Box 1.
**Data source location**	Institution: Parc Taulí Hospital Universitari City: Sabadell Country: Spain
**Data accessibility**	With the article
**Related research article**	Javier Labad, Itziar Montalvo, Alexandre González-Rodríguez, Clemente García-Rizo, Benedicto Crespo-Facorro, José Antonio Monreal, Diego Palao. Pharmacological treatment strategies for lowering prolactin in people with a psychotic disorder and hyperprolactinaemia: a systematic review and meta-analysis. Schizophrenia Research. 2020.

**Value of the data**

These data describe the scientific evidence published between 1980 and 2020 on four pharmacologic strategies (switching antipsychotic treatment, adding aripiprazole, adding other dopamine agonists [e.g., cabergoline, bromocriptine], or adding metformin) for reducing prolactin concentrations in people with psychotic disorders and hyperprolactinaemia.
The data will be useful for psychiatrists, endocrinologists and other health professionals treating patients with psychotic disorders who may develop hyperprolactinaemia.
The data is useful for being considered in future guidelines for treating hyperprolactinaemia in patients with psychotic disorders who usually require long-term antipsychotic treatment that might increase prolactin levels.

## Data description

The data presented in this paper describe supplementary material to the original article [1]. Data will be described in the same order of appearance in the text of the article [1].

Table 1 represents the PICO scheme of the systematic review.

**Table 1:** PICO scheme of the systematic review.

Patient, population or Problem	Intervention	Comparison	Outcome
***What are the characteristics of the patients or population?***	***What interventions are we considering?***	***What is the alternative to the intervention?***	***What are the relevant outcomes?***
Psychotic disorders, including schizophrenia, bipolar disorder and schizophrenia-spectrum psychotic disorders with hyperprolactinaemia.	Four strategies for lowering prolactin: Switching antipsychotics Adding aripiprazole Adding other dopamine agonists (e.g., cabergoline, bromocriptine) Adding metformin	Placebo or maintaining antipsychotic treatment (randomized clinical trials with comparison arms will be included in the meta-analysis). Uncontrolled studies (with comparator) will be included in the systematic review if there is information regarding changes in prolactin with the strategy.	Reduction in prolactin plasma concentrations.

Table 2 comprises the PRISMA Checklist and references all items and pages in the original article.

**Table 2:** PRISMA Checklist of the systematic review.

Section/topic	#	Checklist item	Reported on page #
Title			
Title	1	Identify the report as a systematic review, meta-analysis, or both.	1
Abstract			
Structured summary	2	Provide a structured summary including, as applicable: background; objectives; data sources; study eligibility criteria, participants, and interventions; study appraisal and synthesis methods; results; limitations; conclusions and implications of key findings; systematic review registration number.	1
Introduction			
Rationale	3	Describe the rationale for the review in the context of what is already known.	1–2
Objectives	4	Provide an explicit statement of questions being addressed with reference to participants, interventions, comparisons, outcomes, and study design (PICOS).	2 Table 1 DIB
Methods			
Protocol and registration	5	Indicate if a review protocol exists, if and where it can be accessed (e.g., Web address), and, if available, provide registration information including registration number.	2
Eligibility criteria	6	Specify study characteristics (e.g., PICOS, length of follow-up) and report characteristics (e.g., years considered, language, publication status) used as criteria for eligibility, giving rationale.	2
Information sources	7	Describe all information sources (e.g., databases with dates of coverage, contact with study authors to identify additional studies) in the search and date last searched.	2
Search	8	Present full electronic search strategy for at least one database, including any limits used, such that it could be repeated.	2
Study selection	9	State the process for selecting studies (i.e., screening, eligibility, included in systematic review, and, if applicable, included in the meta-analysis).	2 Fig. 1 DIB
Data collection process	10	Describe method of data extraction from reports (e.g., piloted forms, independently, in duplicate) and any processes for obtaining and confirming data from investigators.	2
Data items	11	List and define all variables for which data were sought (e.g., PICOS, funding sources) and any assumptions and simplifications made.	2
Risk of bias in individual studies	12	Describe methods used for assessing risk of bias of individual studies (including specification of whether this was done at the study or outcome level), and how this information is to be used in any data synthesis.	3
Summary measures	13	State the principal summary measures (e.g., risk ratio, difference in means).	3
Synthesis of results	14	Describe the methods of handling data and combining results of studies, if done, including measures of consistency (e.g., I^2^) for each meta-analysis.	3
Section/topic	#	Checklist item	Reported on page #
Risk of bias across studies	15	Specify any assessment of risk of bias that may affect the cumulative evidence (e.g., publication bias, selective reporting within studies).	3
Additional analyses	16	Describe methods of additional analyses (e.g., sensitivity or subgroup analyses, meta-regression), if done, indicating which were pre-specified.	3
RESULTS			
Study selection	17	Give numbers of studies screened, assessed for eligibility, and included in the review, with reasons for exclusions at each stage, ideally with a flow diagram.	Fig. 1 DIB
Study characteristics	18	For each study, present characteristics for which data were extracted (e.g., study size, PICOS, follow-up period) and provide the citations.	3, List 1 DIB, Tables OA
Risk of bias within studies	19	Present data on risk of bias of each study and, if available, any outcome level assessment (see item 12).	4 Table 9 DIB
Results of individual studies	20	For all outcomes considered (benefits or harms), present, for each study: (a) simple summary data for each intervention group (b) effect estimates and confidence intervals, ideally with a forest plot.	4, Tables & Figure OA
Synthesis of results	21	Present results of each meta-analysis done, including confidence intervals and measures of consistency.	4
Risk of bias across studies	22	Present results of any assessment of risk of bias across studies (see Item 15).	Table 9 DIB
Additional analysis	23	Give results of additional analyses, if done (e.g., sensitivity or subgroup analyses, meta-regression [see Item 16]).	4
Discussion			
Summary of evidence	24	Summarize the main findings including the strength of evidence for each main outcome; consider their relevance to key groups (e.g., healthcare providers, users, and policy makers).	6
Limitations	25	Discuss limitations at study and outcome level (e.g., risk of bias), and at review-level (e.g., incomplete retrieval of identified research, reporting bias).	7–8
Conclusions	26	Provide a general interpretation of the results in the context of other evidence, and implications for future research.	8
Funding			
Funding	27	Describe sources of funding for the systematic review and other support (e.g., supply of data); role of funders for the systematic review.	Role funding source OA

**Fig. 1:**
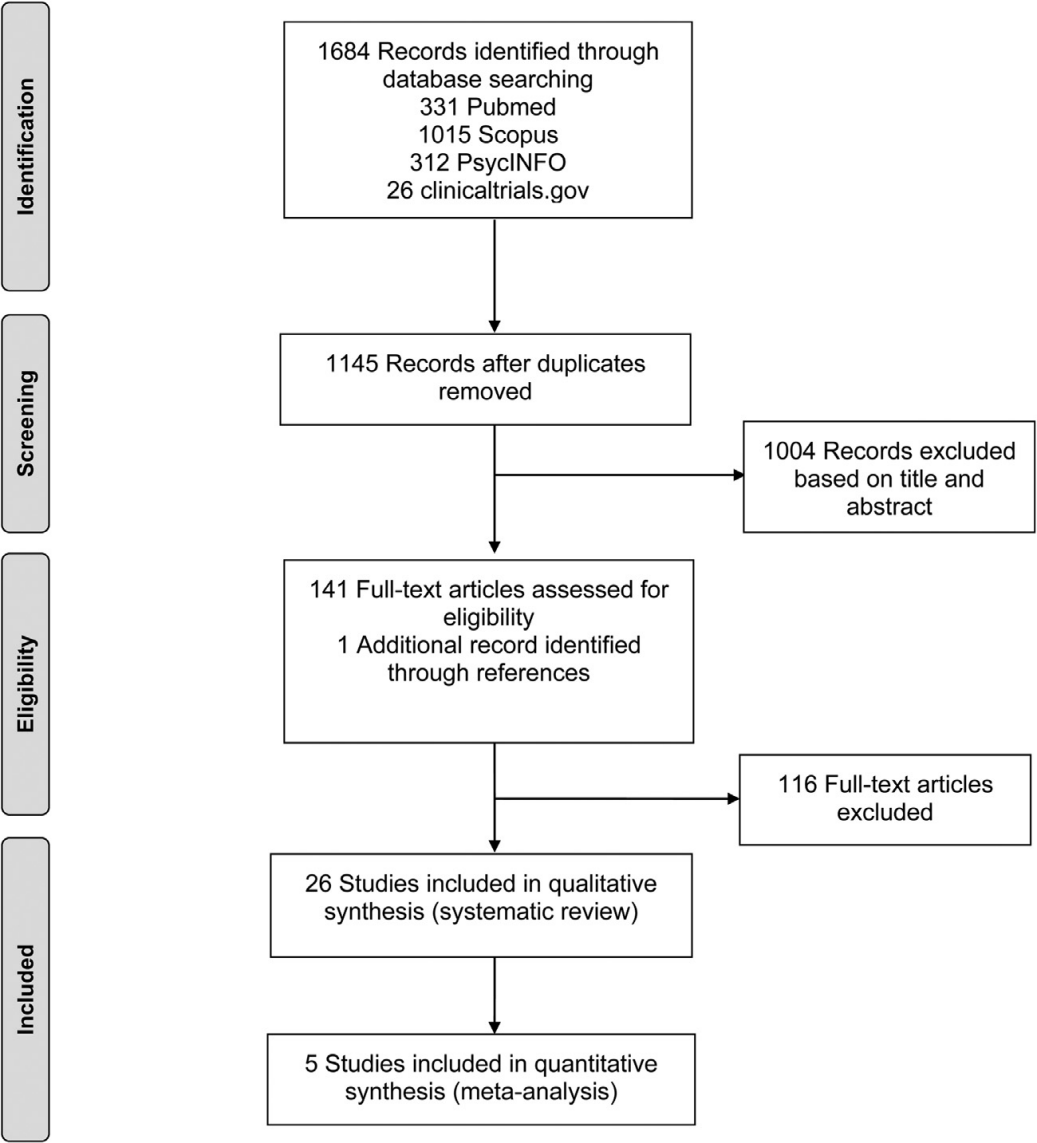
Flow diagram of included studies.

Pages refer to the original article [1]; Abbreviations: OA= Original article; DIB= Data in Brief.Fig. 1. Flow diagram of included studies.

Fig. 1 represents the flow diagram of included studies.

Box 1 explains the procedure for obtaining prolactin concentrations from figures when information on the text or tables was not available.

Code 1 indicates the R code used for calculating the foster plot and funnel plot of the meta-analysis dealing with the primary outcome (prolactin reduction).

Code 2 indicates the R code used for calculating the foster plot and funnel plot of the sensitivity meta-analysis including only placebo-controlled RCTs dealing with the primary outcome (prolactin reduction).

List 1 describes all the studies included in the systematic review.

Tables 3, 4 and 5 show raw data on selected articles dealing with socio-demographic and clinical characteristics (Table 3: Switching studies; Table 4: Aripiprazole addition studies; Table 5: Studies focused on the addition of other dopamine agonists).

**Table 3:** Socio-demographic and clinical characteristics of the samples of studies dealing with switching antipsychotic treatment to lower prolactin.

Reference	Country	Ethnicity	Age (years) Mean (SD)	Duration of illness (years) Mean (SD)	Duration of treatment with PRL-elevating antipsychotic	Substance use	Symptoms (baseline visit)
**Switch to aripiprazole**							
Yoon et al., 2016	Korea	NR	34.7 (8.0)	8.4 (7.4)	>1 month	substance abuse or dependence were excluded	stable over 2 months (no changes in CGI-S)
Chen et al. 2011	China	NR	48.3 (8.2)	17.9 (9.1)	NR	NR	CGI-S: 5.1 (0.9)
Lu et al., 2008	China (Taiwan)	NR	31.7 (9.3)	5.5 (5.4)	NR	NR	CGI-S: 3.4 (0.5) PANSS total: 53.4 (7.7)
Lee et al., 2006	Korea	NR	35.0 (7.4)	2.6 (2.3)	>2 months 6.1 (1.8)	NR	PANSS total: 45.0 (9.0)
**Switch to olanzapine**							
Kinon et al., 2006	USA	48.1% caucasian 40.7% african 7.4% asian 3.7% hispanic	40.0 (10.7)	14.3 (7.9)	NR†	Alcohol or substance abuse were excluded	CGI-S: 3.4 (0.7) PANSS total: 57.5 (16.0)
**Switch to quetiapine**							
Nakajima et al., 2005	Japan	NR	55.0 (12.0)	26.6 (11.6)	>1 month	NR	PANSS positive: 12.8 (4.7) PANSS negative: 18.7 (7.0) PANSS general psychopathology: 41.2 (9.9)
Takahashi et al., 2003	Japan	NR	25.7 (5.1)	NR	14.6 (5.3)	NR	BPRS: 42.4 (7.6)
**Switch to paliperidone**							
Montalvo et al., 2013	Spain	NR	26.6 (5.9)	<5 years (early psychosis)	>6 months	NR	PANSS positive: 10.2 (3.5) PANSS negative: 14.2 (4.4) PANSS general psychopathology: 25.5 (4.5)
**Switch to blonanserin**							
Kawabe et al., 2013	Japan and South Korea	NR	53.9 (8.4)	31.7 (9.4)	NR	NR	BPRS: 47.8 (8.4)

Abbreviations: SD= Standard deviation; NR= Not reported; CGI-*S*= Clinical Global Impression-Severity scale; PANSS= Positive and Negative Syndrome Scale; BPRS= Brief Psychiatric Rating Scale.

† In the study by Kinon et al. (2006), patients who were switched to olanzapine had a mean (SD) duration of previous drug therapy of 11.9 (8.4) years. However, it was not specified the duration of the last antipsychotic drug treatment.

**Table 4:** Socio-demographic and clinical characteristics of the samples of studies dealing with the addition of aripiprazole to lower prolactin.

Reference	Country	Ethnicity	Age (years) Mean (SD)	Duration of illness (years) Mean (SD)	Duration of treatment with PRL-elevating antipsychotic	Substance use	Symptoms (baseline visit)
Kelly et al., 2018	USA	66% african american 33% white	37.8 (8.9)	NR^†^	>2 months	Substance abuse was an exclusion criterion	Clinical stability (psychiatrist consideration) was an inclusion criterion BPRS: 35.4 (10.0) CGI-S: 3.8 (0.9)
Fujioi et al., 2017	Japan	NR	41.3 (8.0)	15.2 (8.3)	>2 months	NR	BPRS: 44.1 (12.5) CGI-S: 4.0 (0.9)
Yoon et al., 2016	Korea	NR	35.8 (7.1)	11.1 (7.3)	>1 month	Substance abuse or dependence was an exclusion criterion	Clinically stable over 2 months (no changes in CGI-S)
Qiaio et al., 2016	China	NR	34.1 (7.0)	14.3 (20.9)	1 month	Substance dependence was an exclusion criterion	PANSS total between 60 and 120 (inclusion criterion) PANSS total: 66.8 (14.2)
Chen et al., 2015	China	NR	34.4 (8.8)	10.38 (7.0)	>6 weeks 9.2 (3.3) weeks	Substance use was an exclusion criterion	Clinical stability (PANSS<70) was an inclusion criterion
Zhao et al., 2015	China	NR	28.9 (7.8)	4.2 (4.2) months	>2 months	Substance use (including alcohol consumption) was an exclusion criterion	Stable psychiatric condition was an inclusion criterion PANSS total: 57.5 (13.5)
Ziadi Trives et al., 2013	Spain	100% white	42.0 (11.4)	NR	>6 months	Substance use was an exclusion criterion	Clinical stability (no changes in treatment or ER visits in the previous 6 months) was an inclusion criterion CGI-S: 3.7 (1.3)
Van Kooten et al., 2011	Netherlands	83.3% white 16.6% asian	47.6 (13.6)	21.3 (12.2)	>12 months 43.2 (36) months	NR	NR
Yasui-Furukori et al., 2010	Japan		44.5 (9.5)	18.9 (13.4)	>3 months	NR	PANSS total: 72.9 (25.0) PANSS positive: 11.9 (3.5) PANSS negative: 19.6 (5.2)
Chen et al., 2010	China (Taiwan)	NR	37.4 (9.0)	12.6 (9.1)	>1 month 9.3 (10.4)	NR	PANSS total: 90.2 (57.2)
Chen et al., 2009	China	NR	NR	NR	NR	NR	NR
Shim et al., 2007	Korea	NR	38.2 (5.3)	15.3 (6.1)	>3 months	Substance use was an exclusion criterion	Clinical stability was an inclusion criterion BPRS: 45–5 (12.3) CGI-S: 4.2 (0.9)

Abbreviations: SD= Standard deviation; NR= Not reported; CGI-*S*= Clinical Global Impression-Severity scale; PANSS= Positive and Negative Syndrome Scale; BPRS= Brief Psychiatric Rating Scale; ER= Emergency room.

**Table 5:** Socio-demographic and clinical characteristics of the samples of studies dealing with the addition of dopamine agonists to lower prolactin.

Reference	Country	Ethnicity	Age (years) Mean (SD)	Duration of illness (years) Mean (SD)	Duration of treatment with PRL-elevating antipsychotic	Substance use	Symptoms (baseline visit)
**Cabergoline addition**							
Kalkavoura et al., 2013	Greece	NR	43.6 (9.8)	21.8 (11.9)	NR†	Alcohol abuse was an exclusion criterion.	PANSS total: 62.9 (2.5)
Coronas et al., 2012	Spain	NR	31.2 (5.0)	NR	NR	NR	Clinical stable before starting cabergoline BPRS: 21.7 (4.9)
Cavallaro et al., 2004	Italy	NR	33.7 (5.6)	NR	>6 months	NR	NR
**Bromocriptine addition**							
Yuan et al., 2008	China	NR	31.1 (7.9)	3.2 (3.5)	>6 months	NR	NR
Bliesener et al., 2004	Germany	NR	20–45 years	NR	>4 months	NR	NR
**Terguride addition**							
Hashimoto et al., 2014	Japan	NR	42.9 (10.6)	15.0 (5.8)	26.7 (11.9) months	NR	NR

Abbreviations: SD= Standard deviation; NR= Not reported; CGI-*S*= Clinical Global Impression-Severity scale; PANSS= Positive and Negative Syndrome Scale; BPRS= Brief Psychiatric Rating Scale.

† In the study Kalkavoura et al. (2013), patients were under antipsychotic treatment for at least 5 years before the inclusion in the study. However, it was not specified the duration of the last antipsychotic drug treatment.

Tables 6, 7 and 8 show raw data on diagnostic criteria for psychotic disorders and exclusion criteria related to reproductive or medical conditions that can alter prolactin levels (Table 6: Switching studies; Table 7: Aripiprazole addition studies; Table 8: Studies focused on the addition of other dopamine agonists).

**Table 6:** Diagnostic criteria for psychotic disorders and exclusion criteria for conditions that can alter prolactin levels of studies dealing with switching antipsychotics to lower prolactin.

Reference	Diagnostic criteria for psychotic disorders	Exclusion criteria (reproductive or medical conditions that can alter prolactin levels)†
**Switch to aripiprazole**		
Yoon et al., 2016	DSM-IV	pregnant or lactating, other diseases that can elevate the prolactin level such as Cushing disease, primary hypothyroidism, liver cirrhosis, renal failure, or prolactinoma
Chen et al. 2011	NR	NR
Lu et al., 2008	DSM-IV	endocrine disease, gynecological problems, or other major medical illnesses .
Lee et al., 2006	DSM-IV	medical maladies (e.g., thyroid or gynecological diseases)
**Switch to olanzapine**		
Kinon et al., 2006	DSM-IV	suspicious MRI scan, pregnant or nursing, bilateral oophorectomy or hysterectomy during or preceding their peri‑menopause, treatment with reproductive hormone therapy, serious unstable illnesses (hepatic, renal, gastroenterologic, respiratory, cardiovascular, endocrinologic, neurologic, immunologic, or hematologic disease), seizures, current agranulocytosis, any other medication with primarily central nervous system activity or that would elevate prolactin
**Switch to quetiapine**		
Nakajima et al., 2005	DSM-IV	NR
Takahashi et al., 2003	DSM-IV	NR
**Switch to paliperidone**		
Montalvo et al., 2013	NR	NR
**Switch to blonanserin**		
Kawabe et al., 2013	DSM-IV	NR

Abbreviations: DSM-IV= Diagnostic and Statistical Manual of Mental Disorders – IV edition; NR= Not reported.

† Information regarding substance use and exclusion criteria due to substance use disorders has been included in Table 3.

**Table 7:** Diagnostic criteria for psychotic disorders and exclusion criteria for conditions that can alter prolactin levels of all studies dealing with the addition of aripiprazole to lower prolactin.

Reference	Diagnostic criteria for psychotic disorders	Exclusion criteria (reproductive or medical conditions that can alter prolactin levels)†
**Aripiprazole addition**		
Kelly et al., 2018	DSM-IV	postmenopause, pregnancy or current post-pregnancy lactation, history of a pituitary tumor (microadenoma, macroadenoma, neoplasm) or Cushing disease, medications that may affect prolactin or cause sexual dysfunction through dopaminergic effects (eg, metoclopramide, methyldopa, reserpine, amoxapine, droperidol, prochlorperazine, promethazine, bromocriptine, and cabergoline)
Fujioi et al., 2017	DSM-IV	menopause, pregnancy, or breast-feeding
Yoon et al., 2016	DSM-IV	pregnant or lactating; other diseases that can elevate the prolactin level such as Cushing disease, primary hypothyroidism, liver cirrhosis, renal failure, or prolactinoma
Qiaio et al., 2016	DSM-IV	neurologic disorder, severe head trauma, or any unstable medical condition
Chen et al., 2015	DSM-IV	significant medical illnesses, such as liver or renal dysfunction, cardiovascular disease, organic brain disorder; pregnant or lactating; other medications than risperidone, anticholinergics or benzodiazepines, such as other antipsychotics, antidepressants, or mood stabilizers etc., which may alter prolactin levels
Zhao et al., 2015	DSM-IV	significant illnesses including severe cardiovascular, hepatic, or renal disease; history of immunosuppression; current or recent radiation or chemotherapy treatment for cancer; pregnancy or breastfeeding; other conditions (e.g., thyroid or gynecological diseases) that could affect serum prolactin levels
Ziadi Trives et al., 2013	NR	intercurrent illness(es) that affect sexual function; other antipsychotics than risperidone; drugs that increase prolactin levels during the 6 months before their inclusion in the study (such as selective serotonin reuptake inhibitors), or any other treatment able to interfere with the adenohypophyseal system (oral contraceptives, tricyclic antidepressants, venlafaxine, mood stabilizers, antihypertensives, or H2 receptors’ antagonists)
Van Kooten et al., 2011	DSM-IV	tuberous sclerosis‡
Yasui-Furukori et al., 2010	DSM-IV	oral contraceptives or estrogen supplemental therapy
Chen et al., 2010	DSM-IV	NR
Chen et al., 2009	DSM-IV	NR
Shim et al., 2007	DSM-IV	medical and/or neurological illness

Abbreviations: DSM-IV= Diagnostic and Statistical Manual of Mental Disorders – IV edition; NR= Not reported.

† Information regarding substance use and exclusion criteria due to substance use disorders has been included in Table 4.

‡ Although no exclusion criteria for medical conditions were reported in the study, the authors specify that one patient with tuberous sclerosis was removed from the study because it could not be excluded that the tuberous sclerosis produced the prolactin elevation.

**Table 8:** Diagnostic criteria for psychotic disorders and exclusion criteria for conditions that can alter prolactin levels of all studies dealing with the addition of dopamine agonists to lower prolactin.

Reference	Diagnostic criteria for psychotic disorders	Exclusion criteria (reproductive or medical conditions that can alter prolactin levels)†
**Cabergoline addition**		
Kalkavoura et al., 2013	DSM-IV	medication implicated in the increase of PRL levels (tricyclic antidepressants, selective serotonin reuptake inhibitor, monoamine oxidase, a-methyldopa, estrogens, oral contraceptives), patients receiving medication implicated in sexual dysfunction (antihypertensives, diuretics, hormones, antifungoral therapy, Parkinson's disease), patients with hyperprolactinaemia due to pregnancy or breastfeeding, hypothyroidism, Cushing's syndrome, cirrhosis, renal failure, meningioma, craniopharyngioma, sarcoidosis, autoimmune disease, tumors of the hypothalamus, acromegalic dysplasia, prolactinoma, diabetes mellitus
Coronas et al., 2012	DSM-IV	NR
Cavallaro et al., 2004	DSM-IV	NR
**Bromocriptine addition**		
Yuan et al., 2008	ICD-10	NR
Bliesener et al., 2004	NR	NR§
**Terguride addition**		
Hashimoto et al., 2014	DSM-IV	NR

Abbreviations: DSM-IV= Diagnostic and Statistical Manual of Mental Disorders – IV edition; ICD-10= International Classification of Diseases – 10th revision; NR= Not reported.

† Information regarding substance use and exclusion criteria due to substance use disorders has been included in Table 5.

§ Although no exclusion criteria for medical condition were reported in the study, the authors specify that all patients had normal hepatic, renal and thyroid function.

Table 9 report the risk of bias summary of all five randomized clinical trials evaluating the addition of aripiprazole for lowering prolactin.

**Table 9:** Risk of bias summary of all five randomized clinical trials evaluating the addition of aripiprazole for lowering prolactin.

**Bias**	Shim et al., 2007	Chen et al., 2015	Zhao et al., 2015	Qiao et al., 2016	Kelly et al., 2018
Random sequence generation	unclear	low	unclear	unclear	low
Allocation concealment	unclear	low	unclear	unclear	low
Blinding of participants and researchers†	low	low	low	low	low
Blinding of outcome assessment†	low	low	low	low	low
Incomplete outcome data	low	low	low	low	low
Selective reporting	low	low	low	low	low
Other bias	low	low	low	low	low
**Quality of the clinical trial**	fair	good	fair	fair	good

† As primary outcome was change in plasma concentrations of prolactin, all randomized clinical trials were considered to have a low risk of bias with respect to the outcome, as this is an objective measure unlikely to be biased even in unblinded situations.

**Fig. 2:**
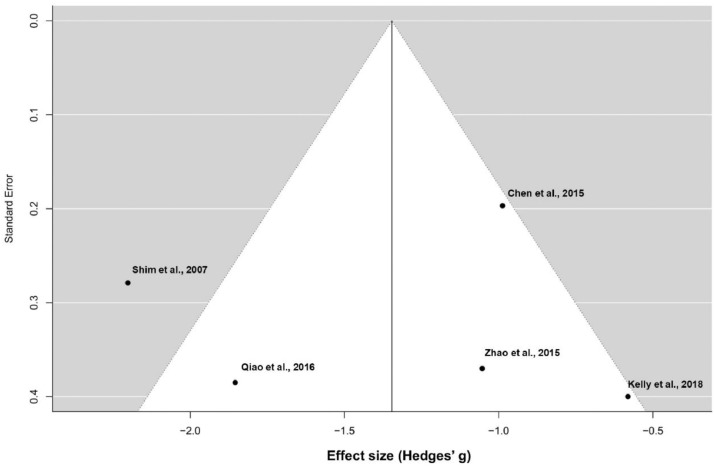
Funnel plot (meta-analysis of prolactin reduction).

Fig. 2 represents the funnel plot of the meta-analysis exploring changes in prolactin concentrations with the addition of aripiprazole. This figure has been generated with R.

**Fig. 3:**
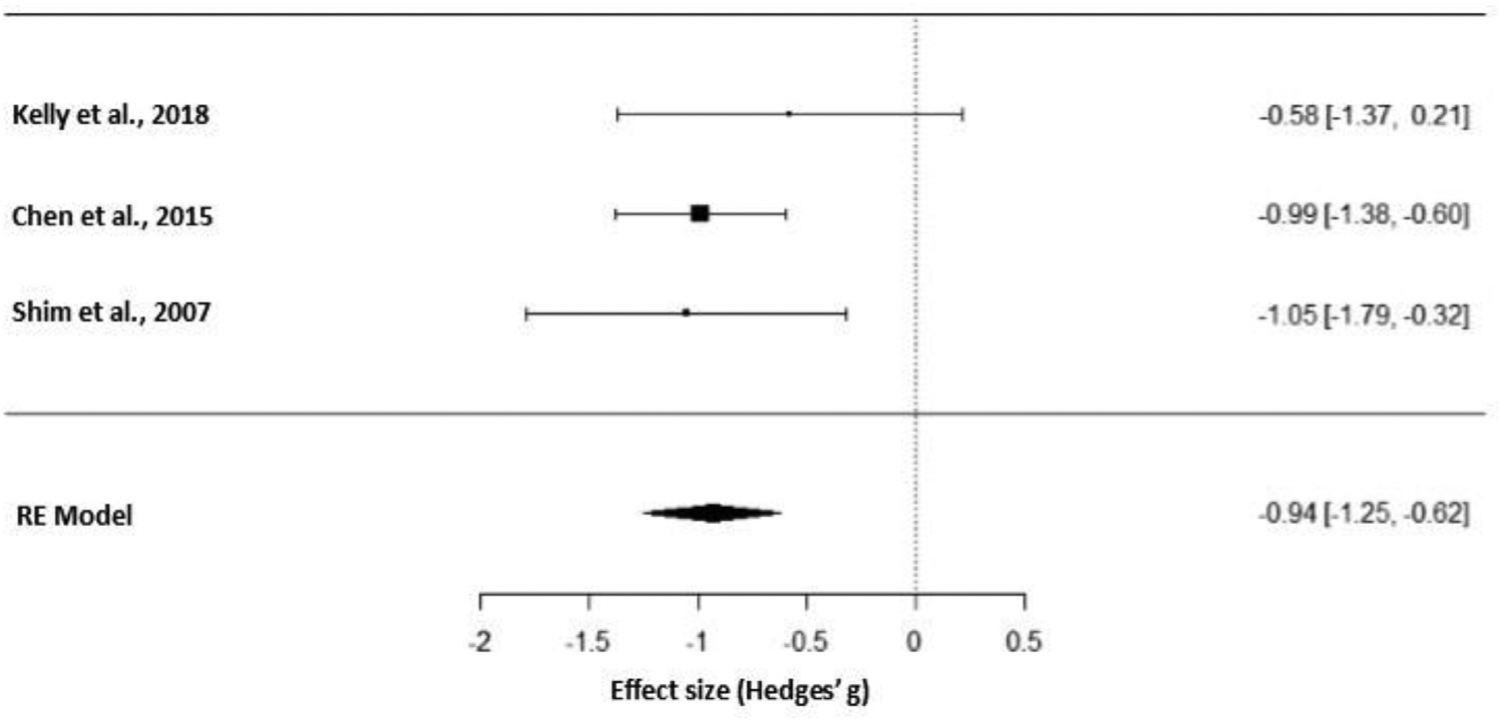
Forest plot of the sensitivity meta-analysis of placebo-controlled RCTs studying adjunctive aripiprazole.

Fig. 3 represents the forest plot of the sensitivity meta-analysis including only placebo-controlled RCTs that studied the effects of adjunctive aripiprazole on prolactin reduction. This figure has been generated with R.

**Fig. 4:**
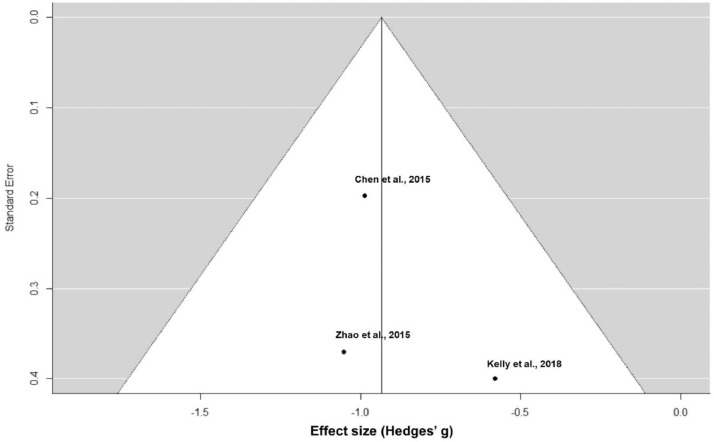
Funnel plot of the sensitivity meta-analysis of placebo-controlled RCTs for prolactin reduction.

Fig. 4 represents the funnel plot of the sensitivity meta-analysis including only placebo-controlled RCTs for prolactin reduction. This figure has been generated with R.

**Fig. 5:**
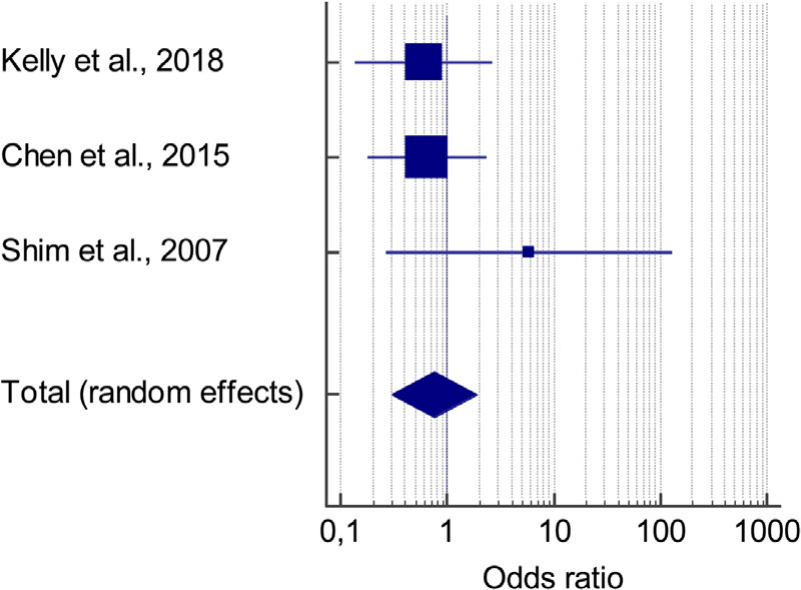
Meta-analysis of withdrawal rates in placebo-controlled RCTs studying adjunctive aripiprazole.

Fig. 5 represents the forest plot of the meta-analysis of withdrawal rates in placebo-controlled RCTs studying adjunctive aripiprazole. This figure has been generated with MedCalc.

Table 10 describes the data regarding the meta-analysis of withdrawal rates in placebo-controlled RCTs studying adjunctive aripiprazole. This table has been generated with MedCalc.

**Table 10:** Data regarding the meta-analysis of withdrawal rates in placebo-controlled RCTs studying adjunctive aripiprazole.

Study	Intervention	Controls	Odds ratio	95% CI	z	P	Weight (%)	
							Fixed	Random
Kelly et al., 2018	4/24	5/20	0,600	0,137 to 2624			39,07	39,07
Chen et al., 2015	8/89	4/30	0,642	0,179 to 2307			51,99	51,99
Shim et al., 2007	2/26	0/28	5816	0,266 to 127,069			8,94	8,94
Total (fixed effects)	14/139	9/78	0,841	0,346 to 2045	−0,382	0,702	100,00	100,00
Total (random effects)	14/139	9/78	0,761	0,303 to 1915	−0,579	0,562	100,00	100,00
**Test for heterogeneity**								
Q	1,8826							
DF	2							
Significance level	*P* = 0,3901							
I^2^ (inconsistency)	0,00%							
95% CI for I^2^	0,00 to 96,44							

**Fig. 6:**
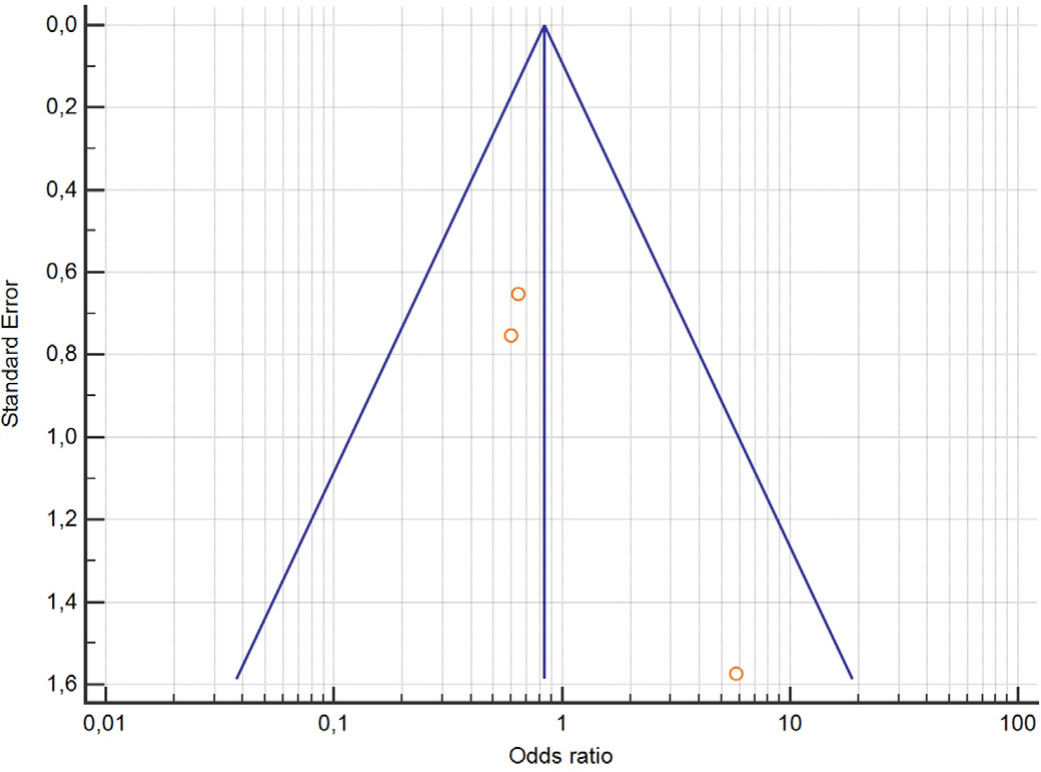
Funnel plot of the meta-analysis of withdrawal rates in placebo-controlled RCTs studying adjunctive aripiprazole.

Fig. 6 represents the funnel plot (meta-analysis of withdrawal rates in placebo-controlled RCTs studying adjunctive aripiprazole). This figure has been generated with MedCalc.

## Experimental design, materials, and methods

We conducted a systematic review of 4 electronic bibliographic databases: PubMed, Scopus, PsycINFO and ClinicalTrials.gov. We aimed to identify clinical trials and observational studies considering four therapeutic options for lowering prolactin: switching antipsychotic treatment, adding aripiprazole, adding other dopamine agonists (e.g., cabergoline, bromocriptine), or adding metformin. The following search strategy was used: prolactin AND (switch* OR aripiprazole OR bromocriptine OR cabergoline OR "dopamine agonist" OR metformin) AND (schizophrenia OR schizoaffective OR psychosis OR psychotic OR bipolar*). Language was restricted to those articles written in English, Spanish, German or French. Studies published between January 1980 and March 2020 were considered for inclusion. The protocol was registered in PROSPERO (CRD42018103466). Preferred Reporting Items for Systematic Reviews and Meta-analyses (PRISMA) guidelines [2] were followed.

Studies were only included if they met the following hierarchical inclusion criteria: (a) contained trials including patients with psychotic disorders (schizophrenia, schizoaffective disorder, bipolar disorder, brief psychotic disorder, delusional disorder, or psychotic disorder not otherwise specified) who had hyperprolactinaemia, (b) clinical trials and observational studies (randomized or non-randomized, controlled or uncontrolled, blinded or open-label [the latter defined as a study where both the researchers and participants know which treatment is being administered]) assessing the efficacy of four therapeutic options for lowering prolactin: switching antipsychotic treatment, adding aripiprazole, adding other dopamine agonists (e.g., cabergoline, bromocriptine), or adding metformin, (c) had information on prolactin levels at baseline and after follow-up that would allow the calculation of the effect size for the reduction in prolactin concentrations, (d) were published in peer-reviewed journals, (e) were written in English, French, German or Spanish, and (f) published between January 1980 and March 2020.

Exclusion criteria were as follows: (a) case reports or studies of less than 5 cases, (b) studies assessing prolactin changes in psychotic patients for whom the main reason for the therapeutic strategy was not hyperprolactinaemia (e.g., switching in treatment-resistant patients).

The main outcome for all studies included in the current systematic review was a change in prolactin levels from baseline to the last available follow-up. As a secondary outcome, we also reviewed withdrawal rates related to safety issues for each treatment strategy. These included adverse effects (physical symptoms or worsening in psychopathological symptoms including psychotic relapses) that led to stopping the treatment strategy. Withdrawal was defined as stopping the study or the assigned treatment once the study had begun; withdrawals prior to medication start were not considered.

Titles and/or abstracts of studies retrieved using the search strategy and those from additional sources were screened independently by two review authors to identify studies that met the inclusion criteria outlined above. The full text of these potentially eligible studies was retrieved and independently assessed for eligibility by two review team members. Any disagreement between them over the eligibility of particular studies was resolved through discussion with two additional reviewers.

A standardised, pre-piloted form was used to extract data from the included studies for assessment of the study quality and evidence synthesis. Two review authors extracted data independently, and discrepancies were identified and resolved through discussion (with two additional authors when necessary). Missing data were requested from study authors. For studies in which the primary condition (hyperprolactinaemia) was included along with other conditions but the outcomes were not specified for the subgroup of patients with hyperprolactinaemia, data were requested from study authors before the study was excluded. For studies that reported prolactin measures at an individual level in tables, we computed the mean and SD. If data were available as the median (interquartile range), the mean and SD were estimated as described previously [3]. In a few cases in which data were only available in figures (either as individual concentrations or aggregated with mean and standard deviation), we extracted this information from figures using the procedure explained in Box 1.

**Box 1:** Procedure for obtaining prolactin concentrations from figures.

1. The PDF of the figure in the article was copied from Adobe PDF zooming by 600%. To determine the prolactin concentration of a particular value (y_n_), three positions (in mm) were determined using rules in Microsoft Publisher (zoom view set at 600%):
y_max_: the maximum concentration of the prolactin scale. We annotated the y position of the Publisher y axis rule (in mm).
y_0_: the minimal concentration of the prolactin scale (usually zero). We annotated the y position of the Publisher y axis rule (in mm).
y_n_: the concentration of the prolactin value to be extracted (unknown). We annotated the y position of the Publisher y axis rule (in mm).
2. Calculation of d1 and d2.
d1= y_0_-y_max_ (in mm).
d2= y_0_-y_n_ (in mm).
3. We aimed to calculate a ratio that would reflect the increase in prolactin concentrations for each mm of y axis rule increase.
Ratio PRL/d1= Range of prolactin concentrations (from y_0_ to y_max_)/d1
4. Calculation of prolactin concentration with the following formula:
PRL (y_n_)= d2 x Ratio PRL/d1

As not all studies included in our systematic review were RCTs, we estimated the effect sizes for the change in prolactin concentrations of all types of studies but only conducted a meta-analysis in RCTs that used placebo or maintaining antipsychotic treatment as comparator arms.

In order to determine the effect size in pre-post designs (all types of studies), the Hedges’ g was used. This effect size measure for changes in prolactin after the therapeutic strategy was determined using an on-line calculator that allows the calculation of the effect size for paired samples tests (https://effect-size-calculator.herokuapp.com/#paired-samples-*t*-test). Another effect size measure considered in the systematic review was confidence intervals (lower and upper limits) using Cohen's d. These measures were calculated using pre-post prolactin and standard deviation measures of all studies. Because the correlation between pre- and post-scores is required to impute the standard deviation within groups from the standard deviation of the difference, this correlation needs to be known. However, because these correlations are not always reported by studies, a common practice is to estimate the correlation from related studies. Therefore, if the correlation between pre- and post-treatment prolactin concentrations was available in the article, we used the known value. For other cases, we estimated the value by using 0.18, a correlation coefficient obtained from the Lu et al. study [4].

The meta-analysis for changes in prolactin concentrations (primary outcome) was performed in R with the metafor package using the Hedge's g estimator (Code 1). Pre-test and post-test pooled standard deviations were considered as explained by Morris [5]. A random effects statistical model was used. We evaluated the risk of bias using the Cochrane risk of bias tool and recoded RCTs into three categories regarding quality: good, fair and poor. Heterogeneity among the included studies was assessed with the I² statistic. Publication bias was explored with funnel plots. We did not use tests for funnel plot asymmetry because less than 10 studies were included in the meta-analysis [6]. A sensitivity analysis was also conducted including only placebo-controlled RCTs. We also conducted another meta-analysis exploring withdrawal from the study (secondary outcome) with MedCalc version 18.11 (MedCalc Software bvba), considering odds ratios as the summary measures.    

Code 1. R code used for the meta-analysis of 5 RCTs on the addition of aripiprazole for reducing prolactin concentrations in patients with a psychotic disorder and hyperprolactinaemia.    

library (metafor)    

# Creating data frame for the treatment group. Prolactin concentrations are represented in the same units as they have been published. Data from the Kelly et al. 2018 study was remitted from the authors.    

datT <- data.frame(

m_pre = *c*(89.36,2173.9,90.34,82.13,1406.64),

m_post = *c*(59.97,718.9,32.91,17.66,514.38),

sd_pre = *c*(65.76,807.9,49.79,88.64,682.46),

sd_post = *c*(55.58,291.9,28.93,36.61,427.0),

ni = *c*(24,30,89,24,56),

ri = *c*(0.18,0.18,0.18,0.18,0.18))    

# Creating data frame for the control group. Prolactin concentrations are represented in the same units as they have been published. Data from the Kelly et al. 2018 study was remitted from the authors.    

datC <- data.frame(

m_pre = *c*(76.86,2623.2,91.61,84.63,1493.75),

m_post = *c*(80.96,3419.9,87.72,91.35,2000.69),

sd_pre = *c*(40.94,1541.7,57.88,37.36,576.3),

sd_post = *c*(42.73,2091.8,57.24,45.10,1077.28),

ni = *c*(18,25,89,27,57),

ri = *c*(0.18,0.18,0.18,0.18,0.18))    

datT <- escalc(measure="SMCR", m1i=*m*_post, m2i=*m*_pre, sd1i=sd_pre, ni=ni, ri=ri, data=datT)

datC <- escalc(measure="SMCR", m1i=*m*_post, m2i=*m*_pre, sd1i=sd_pre, ni=ni, ri=ri, data=datC)    

dat <- data.frame(yi = datT$yi - datC$yi, vi = datT$vi + datC$vi)    

# Pooled (Morris).    

sd_pool <- sqrt((with(datT, (ni-1)*sd_pre^2) + with(datC, (ni-1)*sd_pre^2)) / (datT$ni + datC$ni - 2))

dat <- data.frame(yi = metafor:::.cmicalc(datT$ni + datC$ni - 2) * (with(datT, m_post - m_pre) - with(datC, m_post - m_pre)) / sd_pool)

dat$vi <- 2*(1-datT$ri) * (1/datT$ni + 1/datC$ni) + dat$yi^2 / (2*(datT$ni + datC$ni))

round(dat, 2)    

# Meta-analysis (Random Effects, Hedges g).    

meta<-rma(yi, vi, data=dat, method="HE", digits=2)    

meta

confint(meta)    

# Forest plot.    

forest (meta)    

# Funnel plot.    

funnel (meta,digits=1)      

Code 2. R code used for the sensitivity meta-analysis of 3 placebo-controlled RCTs on the addition of aripiprazole for reducing prolactin concentrations in patients with a psychotic disorder and hyperprolactinaemia.    

library (metafor)    

# Sensitivity meta-analysis including only 3 placebo-controlled RCTs on aripiprazole addition.    

# Creating data frame for the treatment group. Prolactin concentrations are represented in the same units as they have been published. Data from the Kelly et al. 2018 study was remitted from the authors.    

datT <- data.frame(

m_pre = *c*(89.36,90.34,82.13),

m_post = *c*(59.97,32.91,17.66),

sd_pre = *c*(65.76,49.79,88.64),

sd_post = *c*(55.58,28.93,36.61),

ni = *c*(24,89,24),

ri = *c*(0.18,0.18,0.18))    

# Creating data frame for the control group. Prolactin concentrations are represented in the same units as they have been published. Data from the Kelly et al. 2018 study was remitted from the authors.    

datC <- data.frame(

m_pre = *c*(76.86,91.61,84.63),

m_post = *c*(80.96,87.72,91.35),

sd_pre = *c*(40.94,57.88,37.36),

sd_post = *c*(42.73,57.24,45.10),

ni = *c*(18,89,27),

ri = *c*(0.18,0.18,0.18))    

datT <- escalc(measure="SMCR", m1i=*m*_post, m2i=*m*_pre, sd1i=sd_pre, ni=ni, ri=ri, data=datT)

datC <- escalc(measure="SMCR", m1i=*m*_post, m2i=*m*_pre, sd1i=sd_pre, ni=ni, ri=ri, data=datC)    

dat <- data.frame(yi = datT$yi - datC$yi, vi = datT$vi + datC$vi)    

# Pooled (Morris).    

sd_pool <- sqrt((with(datT, (ni-1)*sd_pre^2) + with(datC, (ni-1)*sd_pre^2)) / (datT$ni + datC$ni - 2))

dat <- data.frame(yi = metafor:::.cmicalc(datT$ni + datC$ni - 2) * (with(datT, m_post - m_pre) - with(datC, m_post - m_pre)) / sd_pool)

dat$vi <- 2*(1-datT$ri) * (1/datT$ni + 1/datC$ni) + dat$yi^2 / (2*(datT$ni + datC$ni))

round(dat, 2)    

# Meta-analysis (Random Effects, Hedges g).    

meta<-rma(yi, vi, data=dat, method="HE", digits=2)

meta

confint(meta)    

# Forest plot.    

forest (meta)    

# Funnel plot.    

funnel (meta,digits=1)      

List 1. Studies included in the systematic review (alphabetical order).

**Those studies included in the meta-analysis are indicated in bold**.

1 Bliesener N, Yokusoglu H, Quednow BB, Klingmüller D, Kühn KU. Usefulness of bromocriptine in the treatment of amisulpride-induced hyperprolactinemia: A case report. Pharmacopsychiatry. 2004; 37:189–191.. doi:10.1055/s-2004–827,176
2 Cavallaro R, Cocchi F, Angelone SM, Lattuada E, Smeraldi E. Cabergoline treatment of risperidone-induced hyperprolactinemia: a pilot study. J Clin Psychiatry. 2004;65:187–190. doi:10.4088/JCP.v65n0207
3 Chen CK, Huang YS, Ree SC, Hsiao CC. Differential add-on effects of aripiprazole in resolving hyperprolactinemia induced by risperidone in comparison to benzamide antipsychotics. Prog Neuro-Psychopharmacology Biol Psychiatry. 2010; 34:1495–1499. doi:10.1016/j.pnpbp.2010.08.012
4 Chen CY, Lin TY, Wang CC, Shuai HA. Improvement of serum prolactin and sexual function after switching to aripiprazole from risperidone in schizophrenia: a case series. Psychiatry Clin Neurosci. 2011; 65:95–97. doi:10.1111/j.1440–1819.2010.02156.x
5 **Chen JX, Su YA, Bian QT, et al. Adjunctive aripiprazole in the treatment of risperidone-induced hyperprolactinemia: A randomized, double-blind, placebo-controlled, dose-response study. Psychoneuroendocrinology. 2015;58:130–140. doi:10.1016/j.psyneuen.2015.04.011**
6 Chen JX, Su YA, Wang SL, et al. Aripiprazole treatment of risperidone-induced hyperprolactinemia. J Clin Psychiatry. 2009; 70:1058–1059.. doi:10.4088/JCP.08l04671
7 Coronas R, Cobo J, Gimenez-Palop O, Ortega E, Marquez M. Safety of Cabergoline in the Management of Pituitary Prolactin-Induced Symptoms with Patients Treated with Atypical Neuroleptics. Curr Drug Saf. 2012;7:92–98. doi:10.2174/157,488,612,802,715,753
8 Fujioi J, Iwamoto K, Banno M et al. Effect of Adjunctive Aripiprazole on Sexual Dysfunction in Schizophrenia: A Preliminary Open-Label Study. Pharmacopsychiatry. 2017; 50:74–78. doi:10.1055/s-0042–116,323
9 Hashimoto K, Sugawara N, Ishioka M, Nakamura K, Yasui-Furukori N. The effects of additional treatment with terguride, a partial dopamine agonist, on hyperprolactinemia induced by antipsychotics in schizophrenia patients: A preliminary study. Neuropsychiatr Dis Treat. 2014; 10:1571–1576. doi:10.2147/NDT.S68298
10 Kalkavoura C, Michopoulos I, Arvanitakis P, et al. Effects of Cabergoline on Hyperprolactinemia, Psychopathology, and Sexual Functioning in Schizophrenic Patients. Exp Clin Psychopharmacol. 2013;21:332–341. doi:http://dx.doi.org/10.1037/a0033448
11 Kawabe K, Horiuchi F, Ueno SI. Blonanserin, a novel antipsychotic, is suitable for treating schizophrenia associated with hyperprolactinemia: A case series. Clin Neuropharmacol. 2013; 36:239–241. doi:10.1097/WNF.0000000000000006
12 **Kelly DL, Powell MM, Wehring HJ, et al. Adjunct Aripiprazole Reduces Prolactin and Prolactin-Related Adverse Effects in Premenopausal Women with Psychosis: Results from the DAAMSEL Clinical Trial. J Clin Psychopharmacol. 2018; 38:317–326. doi:10.1097/JCP.0000000000000898**
13 Kinon BJ, Ahl J, Liu-Seifert H, Maguire GA. Improvement in hyperprolactinemia and reproductive comorbidities in patients with schizophrenia switched from conventional antipsychotics or risperidone to olanzapine. Psychoneuroendocrinology. 2006; 31:577–588. doi:10.1016/j.psyneuen.2005.12.006
14 Lee BH, Kim YK, Park SH. Using aripiprazole to resolve antipsychotic-induced symptomatic hyperprolactinemia: A pilot study. Prog Neuro-Psychopharmacology Biol Psychiatry. 2006; 30:714–717. doi:10.1016/j.pnpbp.2006.02.001
15 Lu ML, Shen WW, Chen CH. Time course of the changes in antipsychotic-induced hyperprolactinemia following the switch to aripiprazole. Prog Neuro-Psychopharmacology Biol Psychiatry. 2008; 32:1978–1981. doi:10.1016/j.pnpbp.2008.09.016
16 Montalvo I, Ortega L, López X, et al. Changes in prolactin levels and sexual function in young psychotic patients after switching from long-acting injectable risperidone to paliperidone palmitate. Int Clin Psychopharmacol. 2013; 28:46–49. doi:10.1097/YIC.0b013e32835ba832
17 Nakajima M, Terao T, Iwata N, Nakamura J. Switching female schizophrenic patients to quetiapine from conventional antipsychotic drugs: Effects on hyperprolactinemia. Pharmacopsychiatry. 2005; 38:17–9. doi:10.1055/s-2005–837,766
18 **Qiao Y, Yang F, Li C, et al. Add-on effects of a low-dose aripiprazole in resolving hyperprolactinemia induced by risperidone or paliperidone. Psychiatry Res. 2016; 237:83–89. doi:10.1016/j.psychres.2015.12.033**
19 **Shim JC, Shin JGK, Kelly DL, et al. Adjunctive treatment with a dopamine partial agonist, aripiprazole, for antipsychotic-induced hyperprolactinemia: A placebo-controlled trial. Am J Psychiatry. 2007; 164:1404–1410. doi:10.1176/appi.ajp.2007.06071075**
20 Takahashi H, Higuchi H, Kamata M, et al. Effectiveness of Switching to Quetiapine for Neuroleptic-Induced Amenorrhea. J Neuropsychiatry Clin Neurosci. 2014; 15:375–377. doi:10.1176/jnp.15.3.375
21 Van Kooten M, Arends J, Cohen D. Preliminary report: A naturalistic study of the effect of aripiprazole addition on risperidone-related hyperprolactinemia in patients treated with risperidone long-acting injection. J Clin Psychopharmacol. 2011; 31:126–128. doi:10.1097/JCP.0b013e318205e1aa
22 Yasui-Furukori N, Furukori H, Sugawara N, Fujii A, Kaneko S. Dose-dependent effects of adjunctive treatment with aripiprazole on hyperprolactinemia induced by risperidone in female patients with schizophrenia. J Clin Psychopharmacol. 2010; 30:596–599. doi:10.1097/JCP.0b013e3181ee832d
23 Yoon HW, Lee JS, Park SJ, et al. Comparing the effectiveness and safety of the addition of and switching to aripiprazole for resolving antipsychotic-induced hyperprolactinemia: A multicenter, open-label, prospective study. Clin Neuropharmacol. 2016; 39:288–294. doi:10.1097/WNF.0000000000000175
24 Yuan HN, Wang CY, Sze CW, et al. A randomized, crossover comparison of herbal medicine and bromocriptine against risperidone-induced hyperprolactinemia in patients with schizophrenia. J Clin Psychopharmacol. 2008; 28:264–370. doi:10.1097/JCP.0b013e318172473c
25 **Zhao J, Song X, Ai X, et al. Adjunctive aripiprazole treatment for risperidone-induced hyperprolactinemia: An 8-week randomized, open-label, comparative clinical trial. PLoS One. 2015; 10:e0139717. doi:10.1371/journal.pone.0139717**
26 Ziadi Trives M, Bonete Llácer J-M, García Escudero M-A, Martínez Pastor CJ. Effect of the addition of aripiprazole on hyperprolactinemia associated with risperidone long-acting injection. J Clin Psychopharmacol. 2013; 33:538–541. doi:10.1097/jcp.0b013e3182970431

## Declaration of Competing Interest

Javier Labad, Benedicto Crespo-Facorro and Clemente García-Rizo have received fees for consultancy or advice services and lecture fees from Janssen-Cilag, Lundbeck and Otsuka Pharmaceuticals.

Although some of the authors have received honoraria for lectures or advisory boards from Otsuka, Lundbeck and Janssen-Cilag, we want to underscore that no drug company has participated in the design or funding of the study.
